# CAN GOOD SELF-RATED HEALTH MITIGATE THE FRAILTY-INDUCED HEALTH CARE EXPENDITURE? EVIDENCE FROM CHINA

**DOI:** 10.1093/geroni/igad104.1449

**Published:** 2023-12-21

**Authors:** Haiyu jin

**Affiliations:** University of Southampton, Southampton, England, United Kingdom

## Abstract

As the pathophysiology of frailty remains unknown, intervention efforts have primarily focused on improving functions and alleviating symptoms instead of treating frailty-causing biology. Frail individuals are not a clinically homogeneous group; further risk stratification may identify subgroups more resilient to health risks. Self-rated health (SRH), a simple measure of an individual’s overall perception of overall health, might be a surrogate of resilience among the frail. This work aims to determine the joint association of frailty and SRH with healthcare expenditure. Data were from the China Health and Retirement Longitudinal Study. Persons were classified as non-frail, prefrail, and frail. SRH was classified as poor (including very poor) and good (fair/good/very good/excellent). Healthcare expenditure was classified into outpatient, inpatient, and self-treatment. The Cox model was used to address the study’s aims. All analyses were adjusted for age, sex, residence, education, income, insurance, smoking, and chronic conditions. Although frailty was positively correlated with poor SRH, 36.0% of the frail reported good health and 26.6% reporting poor health were deemed non-frail. Frailty and SRH were independently associated with higher odds of incurring and higher average expenditures. Among persons with poor SRH, prefrailty and frailty were associated with higher odds of incurring the cost and higher average expenditures than the non-frail, while no association was found between frailty and healthcare expenditure among those with good SRH. Frailty was associated with higher healthcare expenditure only among persons with poor SRH. Frail individuals reporting good health are resilient to frailty-induced health risks.

